# GWAS Central: a comprehensive resource for the comparison and interrogation of genome-wide association studies

**DOI:** 10.1038/ejhg.2013.274

**Published:** 2013-12-04

**Authors:** Tim Beck, Robert K Hastings, Sirisha Gollapudi, Robert C Free, Anthony J Brookes

**Affiliations:** 1 Department of Genetics, University of Leicester, Leicester, UK

**Keywords:** GWAS, Genotype, Phenotype, SNP, Database

## Abstract

To facilitate broad and convenient integrative visualization of and access to GWAS data, we have created the GWAS Central resource (http://www.gwascentral.org). This database seeks to provide a comprehensive collection of summary-level genetic association data, structured both for maximal utility and for safe open access (i.e., non-directional signals to fully preclude research subject identification). The resource emphasizes on advanced tools that allow comparison and discovery of relevant data sets from the perspective of genes, genome regions, phenotypes or traits. Tested markers and relevant genomic features can be visually interrogated across up to 16 multiple association data sets in a single view, starting at a chromosome-wide view and increasing in resolution down to individual bases. In addition, users can privately upload and view their own data as temporary files. Search and display utility is further enhanced by exploiting phenotype ontology annotations to allow genetic variants associated with phenotypes and traits of interest to be precisely identified, across all studies. Data submissions are accepted from individual researchers, groups and consortia, whereas we also actively gather data sets from various public sources. As a result, the resource now provides over 67 million *P*-values for over 1600 studies, making it the world's largest openly accessible online collection of summary-level GWAS association information.

## Introduction

The genome-wide association study (GWAS) era has improved our understanding of disease aetiology by identifying genetic variants associated with complex human traits and disease phenotypes. However, to fully evaluate the data emerging from these studies, researchers need convenient ways to access and visualize the totality of investigations so far completed, while not compromising any individual's privacy or informed consent. To this end, we herein describe GWAS Central, a comprehensive genetic association database, designed to enable multiple study integration via graphical displays and extensive textual content.

Other GWAS depositories, such as dbGaP^1^ (http://www.ncbi.nlm.nih.gov/gap/) and EGA (http://www.ebi.ac.uk/ega/), act as archival systems that provide controlled access to individual-level GWAS data and open access to some categories of summary-level data. This approach is merited, given that it is possible to identify the participation of a research subject within the full range of summary-level data.^2^ Smaller amounts of summary-level GWAS data are available from resources such as the NHGRI GWAS Catalog^3^ and the Open Access Database of Genome-wide Association Results (OADGAR),^4^ with their content being restricted to marker signals that exceed predefined *P*-value thresholds. The semi-arbitrary imposition of such cut-offs is unfortunate, in that it prevents direct comparison across the totality of signals (within and between related studies), in order to identify consistently positive markers.

Convenient, dedicated resources that provide unfettered access to all GWAS summary-level data are therefore needed, powered by user-friendly tools for instant interrogation and visualization of unified views of the data. In particular, such displays also need to incorporate information about the tested markers, such as chromosome location, alleles and 5′ and 3′ flanking sequences of SNPs. Ideally, the investigated phenotypes will be represented by standardized terminologies, thus allowing meaningful cross-study searches to be conducted.

On the basis of the above considerations, we designed and created the GWAS Central resource (http://www.gwascentral.org). Here we describe the ways in which this database enables experimental biologists to explore and compare data in the GWAS domain, from either a genotype or phenotype starting point.

### Implementation

GWAS Central collates association data and study metadata from many disparate sources whose data are available in different formats and to differing degrees of detail. These diverse GWAS data are integrated in a flexible and coherent data model that was described previously in an earlier incarnation of the database, named HGVbaseG2P.^5^ GWAS Central builds upon core genomic variation visualization and comparison concepts from HGVbaseG2P to provide new features, such as downloadable detailed data reports, semantically standardized phenotype ontology searching, optimized data visualizations, private upload and comparison of user data, and tools for remote data interrogation. The various resources collated by GWAS Central include data sets from other sites, such as the NHGRI GWAS Catalog, OADGAR and complete association data sets from the 10 trait-based investigations of the 1958 Birth Cohort.^6^ In addition, a substantial amount of data have also been obtained by directly requesting data from researchers and consortia and from numerous unsolicited data submissions from researchers who wish for their newly published data to be included in GWAS Central. All data submitters are fully acknowledged, with the contributing resources and the original authors of each study cited on the website.

The gathered and submitted data are extensively curated to maximize quality and completeness. This includes checking that all genetic markers have valid dbSNP rs numbers, assessing whether the alleles and strand representation of these are correct, eliminating duplicate markers, combining multiple data sets for discrete studies and populating extensive metadata. In addition, we manually evaluate each study for its range of phenotype content and apply appropriately chosen ontology terms to ensure that the phenotype descriptions are standardized across all studies. In this task, we identify for each phenotype an equivalent or most appropriate term from the National Library of Medicine's MeSH controlled vocabulary. MeSH is used because it offers familiarity to biologists as a result of PubMed MEDLINE indexing and it also provides good generalized descriptions of phenotypes. The Human Phenotype Ontology (HPO) is also used to annotate phenotypes in cases where HPO offers a more specific description.^7^

To allow flexible access and data discovery, GWAS Central queries are structured into three types, namely, genotype, phenotype or keyword orientated. Genotype searches can be based on HGNC gene symbols, genomic region coordinates or dbSNP rs numbers. Phenotype searches are linked to MeSH and HPO annotations, as well as to the original free-text descriptions used in publications. Keyword searches interrogate text contained in study titles and abstracts, PubMed IDs and author names.

## Results

GWAS Central content is organized and displayed using three levels, representing the core aspects of each GWAS (Supplementary Figure 1). These describe the following: 1) the top-level study summary presenting the cohorts used to carry out the study and the association result sets comprising *P*-values, odds ratios, frequency data and list of associated markers; 2) phenotypes observed during each experiment within a study with both free-text descriptions and ontology-derived annotations, along with the phenotyping methods; 3) detailed marker information for those markers identified in the study, such as current genomic coordinates, genic relationships, revision history (eg, if it has been merged/deleted), and its specific sequence information. A summary of each study can be exported in XML and JSON file formats and the ‘top' 100 association results for each experiment per study can be exported as an Excel spreadsheet, in text file (CSV and TSV), news feed (RSS and Atom) and semantic web compatible (RDF) formats.

**Figure 1:**
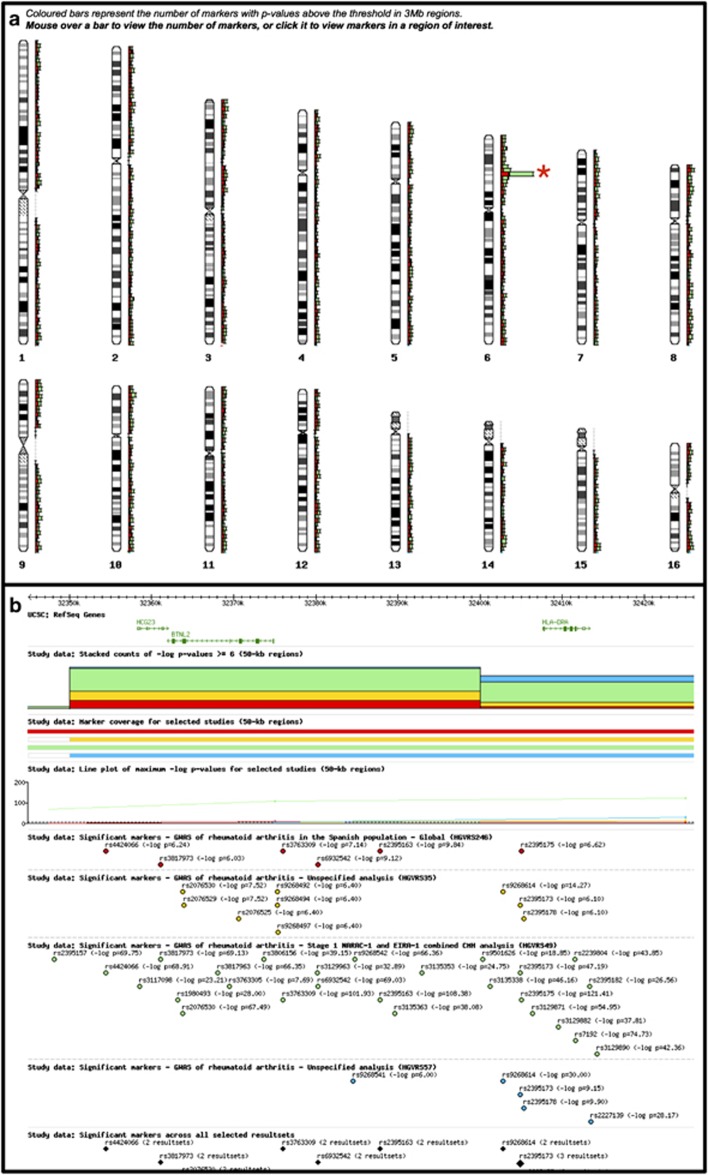
Data integration example using the GWAS Central graphical browser. (**a**) The ‘genome view' of four distinct rheumatoid arthritis studies showing regions of association. The coloured bars denote counts of marker *P*-values below a selected and adjustable threshold. Selecting the peak on chromosome 6 adjacent to the red asterisk zooms in on that section of the genome to give **b**. (**b**) The higher resolution ‘region view' of the data provides UCSC RefSeq genes, HapMap SNPs for the various cohorts and Human Genetic Mutation Database rare disease mutations.

Reflecting the goal of GWAS Central to allow multiple GWAS to be compared, an integrated browser tool allows up to 16 association data sets to be correlated and visually interrogated. In addition, association data can be anonymously uploaded to the resource for comparison alongside data sets contained in the database. The real-time upload feature requires a simple delimiter-separated value file consisting of two columns containing the dbSNP rs number and the associated *P*-value (the online documentation provides further details: http://www.gwascentral.org/info/using-the-browser/custom-upload/). All user data are automatically removed from our servers after 48 h. The browser allows interesting signals to be examined in detail by switching between the ‘genome' view of all chromosomes and the ‘region' view of increasing resolution down to individual nucleotide resolution (Figure 1). Association data can be viewed in the context of genomic features such as genes, HGMD variants, HapMap SNPs and linkage disequilibrium maps. The data in regions selected by the user can also be opened in the UCSC and Ensembl genome browsers or exported in standard BED or GFF file formats for further analysis. Furthermore, all displays and outputs allow *P*-value thresholds to be applied, and hence only those associations a researcher deems significant are presented.

GWAS Central also provides a BioMart-based system,^8^ named GWAS Mart, for advanced data interrogation and deeper data downloads. The standard ‘MartView' interface (http://www.gwascentral.org/biomart/martview) allows flexible querying against the complete database and download of large data sets consisting of either a complete study or up to 1000 markers and associated data per download. Query results can be viewed and exported in HTML and text file (CSV and TSV) formats. Larger data downloads are made available to researchers who agree with GWAS Central's data sharing policy (available at http://www.gwascentral.org/info/data/data-sharing-statement/). In addition, BioMart web services make GWAS Central data available for use in remote data analyses and bioinformatics workflows.

As of September 2013, 1605 studies and 67 723 637 *P*-values are available, corresponding to 2 935 163 unique dbSNP rs numbers. The myriad phenotype terms encompassed by these studies have been additionally grouped into 22 upper-level MeSH disease categories (Supplementary Figure 2), with common areas extensively covered (eg, neoplasms, nutritional and metabolic diseases, nervous system illness and immune system disorders).

## Discussion

GWAS Central provides a valuable toolkit for the storage, mining and display of summary-level association data. This resource is substantially more comprehensive than other openly available projects with a similar focus (ie, tens of millions of *P*-values vs thousands; Table 1). To fully preclude participant re-identification from the open content of this resource, all association signals are presented in a non-directional manner (ie, risk alleles not stated). GWAS Central also provides a range of user tools and interfaces that were not previously available from a single resource. Table 1 compares GWAS Central with other related resources (GWAS Catalog, OADGAR and SNPedia^9^) with regard to the features provided.

Towards the goal of bringing all GWAS data conveniently together, we have collated data from several sources that are not openly available elsewhere; for example, an imminent release will include data from the International Serious Adverse Event Consortium. Further, we have engineered and optimized the software to deal with all available summary-level data generated during a study, instead of limiting the content to only a small number of ‘top' *P*-values.

Researchers have reported that their GWAS publications have received increased attention and citations as a result of inclusion in GWAS Central. We actively encourage researchers to submit their complete summary-level findings to GWAS Central, and to this end we provide an Excel template to help researchers organize and supply single-study data. To further lower the barrier to sharing large data sets or multiple studies, researchers can supply data in whichever format they have available and we will apply our automated conversion pipelines to ensure that the findings are correctly represented in the database (the online documentation provides further details on how to submit data: http://www.gwascentral.org/info/how-to/submit-data/). Researchers are also encouraged to ensure their findings are properly and optimally displayed in GWAS Central, and we will prioritize all requests to modify or update existing data with additional *P*-values (the online documentation provides further details: http://www.gwascentral.org/info/how-to/how-to-modify-data/).

For consortia and research groups that wish to play a more active role in displaying their GWAS findings, we make the source of the GWAS Central platform available as part of a collaboration. Thereby, institutes, consortia, teams and even whole countries, working together on a common area of interest, can serve their data to the research community on their own terms and thereby meet the expectations of funders. Indeed, a group based in India has recently adopted this system (GWAS Central India: http://www.vigeyegpms.in/gwascentralindia/). In time, these implementations may well be federated to allow searching across different data sets. Thus, the provision of local versions of GWAS Central will reduce the effort involved in creating and maintaining GWAS summary-level databases while making important information in the disease genetics field available to the global research community.

## Figures and Tables

**Table 1:** Comparison of features between GWAS Central and related resources

*Resource feature*	*GWAS Central*	*GWAS Catalog*	*OADGAR*	*SNPedia*
Relationships between SNPs and disease	●	●	●	●
Includes *P*-values <1.0 × 10^−5^	●	●	●	●
Includes *P*-values between 0.001 and 1.0 × 10^−5^	●		●	
Includes *P*-values >0.001	●			
Interactive graphical visualization of SNPs	●	●		
Phenotypes searchable using multiple ontologies	●			
Web service access	●			●
Upload and comparison of user's data	●			
Website documentation on how users can submit data	●			●
Unrestricted download	Full study or 1K *P*-values^a^	All data	All data	All data
Number of studies^b^	1605	1688	118	Not stated
Number of *P*-values^b^	67 723 637	13 839	56 411	Not stated

Abbreviations: GWAS, genome-wide association study; OADGAR, Open Access Database of Genome-wide Association Results; SNP, single-nucleotide polymorphism.

a A full study or 1000 *P*-values from multiple studies are available for unrestricted download. All data are available with agreement.

b As of September 2013.
