# Supplementary material for: GWAS Central: a comprehensive resource for the comparison and interrogation of genome-wide association studies
Source: Eur J Hum Genet. 2013 Dec 4;22(7):949–52. doi: 10.1038/ejhg.2013.274 (PMC4060122; doi:10.1038/ejhg.2013.274)
Supplement: Supplementary Figure S1 [file ejhg2013274x1.pdf]

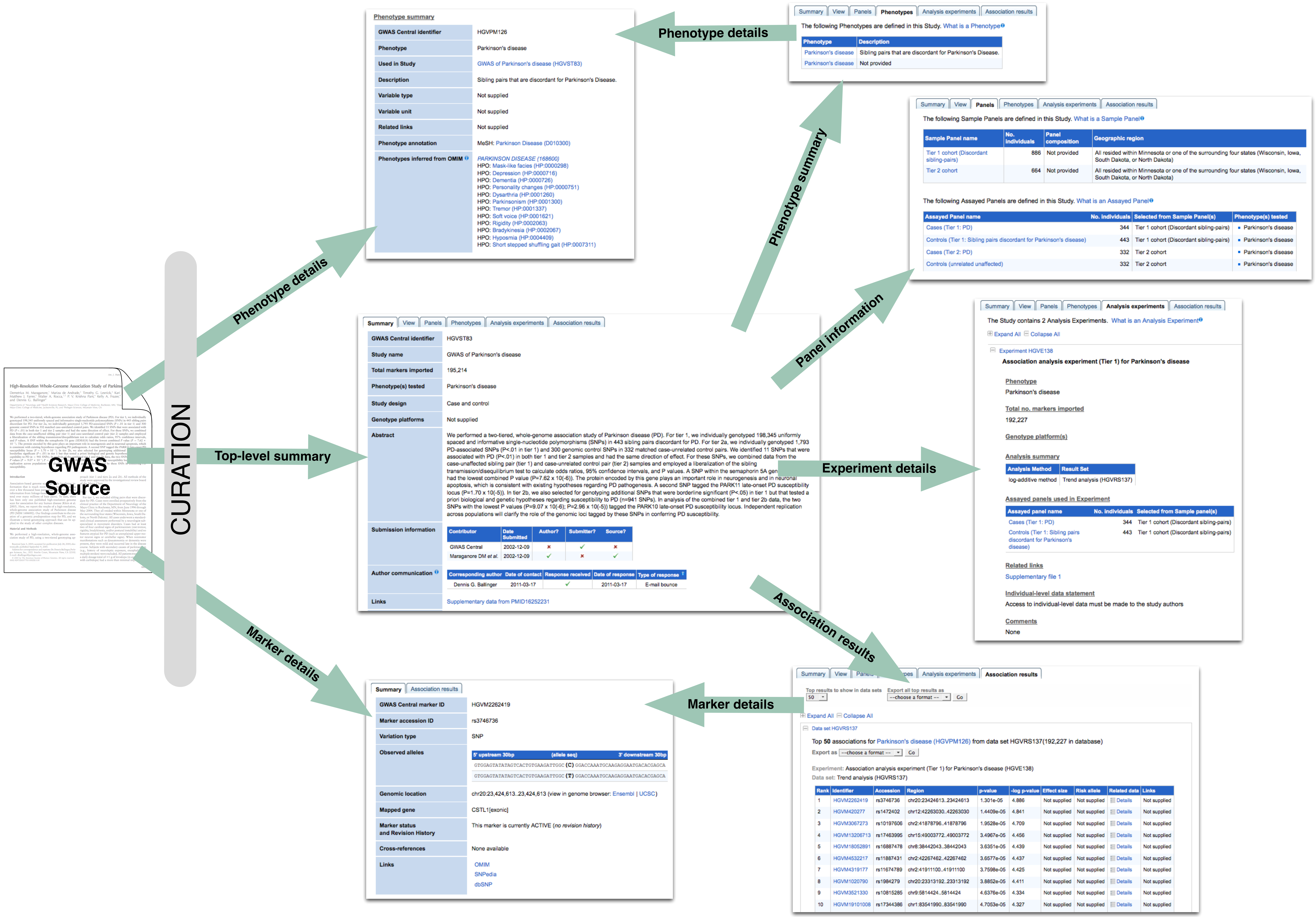

CURATION
GWAS Source
Phenotype details
Top-level summary
Marker details
Phenotype summary
Panel information
Experiment details
Association results
Phenotype details
Marker details
Phenotype summary
GWAS Central identifier HGVPM126
Phenotype Parkinson's disease
Used in Study GWAS of Parkinson's disease (HGVST83)
Description Sibling pairs that are discordant for Parkinson's Disease.
Summary View Panels Phenotypes Analysis experiments Association results
GWAS Central identifier HGVST83
Study name GWAS of Parkinson's disease
Total markers imported 195,214
Phenotype(s) tested Parkinson's disease
Study design Case and control
Genotype platforms Not supplied
Abstract
Submission information
Author communication
Links Supplementary data from PMID16252231
The following Phenotypes are defined in this Study. What is a Phenotype
The following Sample Panels are defined in this Study. What is a Sample Panel
The following Assayed Panels are defined in this Study. What is an Assayed Panel
The Study contains 2 Analysis Experiments. What is an Analysis Experiment
Association analysis experiment (Tier 1) for Parkinson's disease
GWAS Central marker ID HGVM2262419
Marker accession ID rs3746736
Variation type SNP
Observed alleles
Genomic location
Mapped gene CSTL1[exonic]
Top 50 associations for Parkinson's disease (HGVPM126) from data set HGVRS137(192,227 in database)
