# Supplementary figures and images for: GWAS Central: a comprehensive resource for the comparison and interrogation of genome-wide association studies
Source: Eur J Hum Genet. 2013 Dec 4;22(7):949–52. doi: 10.1038/ejhg.2013.274 (PMC4060122; doi:10.1038/ejhg.2013.274)

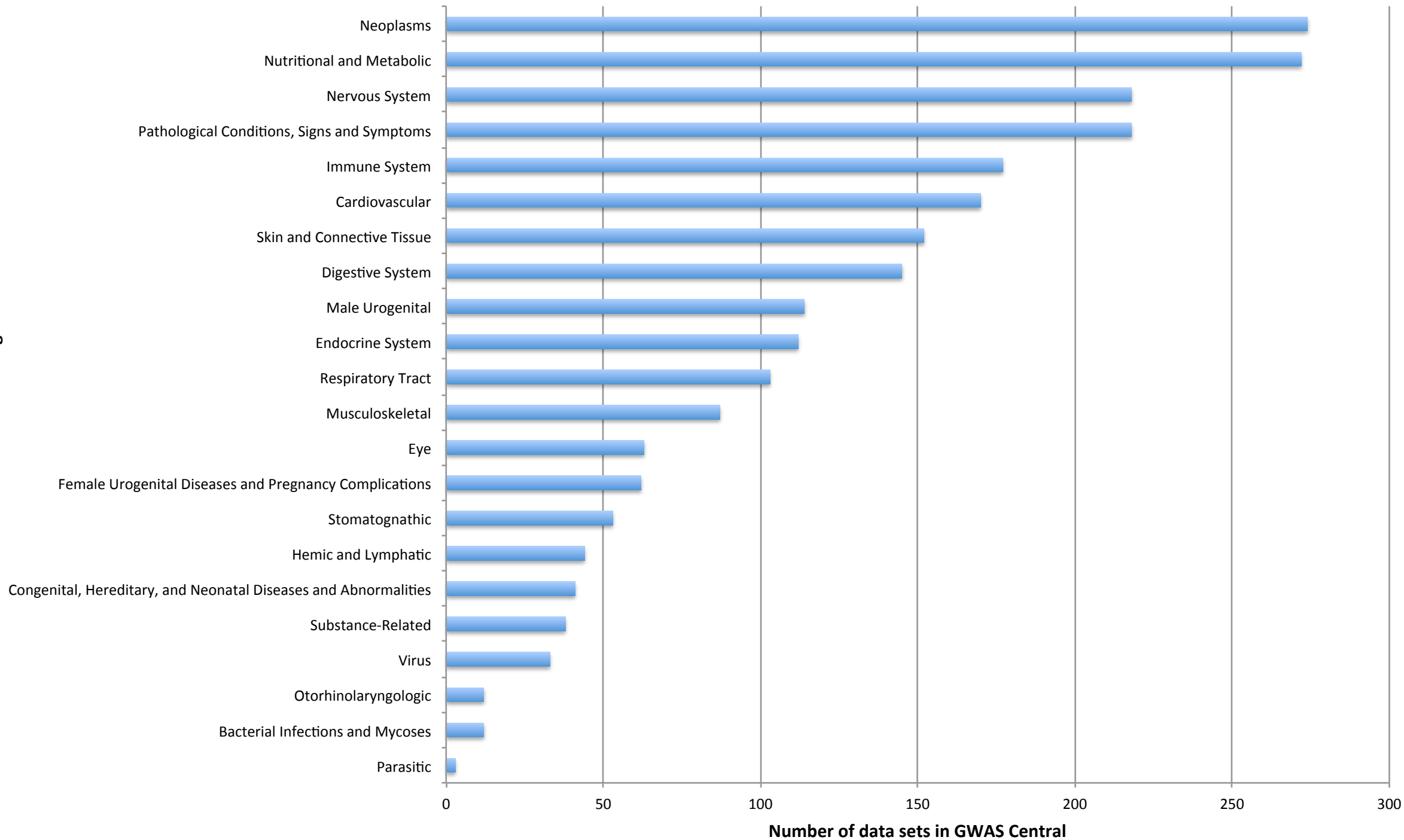

Supplement: Supplementary Figure S2 [file ejhg2013274x2.pdf]
