# Supplementary material for: GWAS Central: a comprehensive resource for the comparison and interrogation of genome-wide association studies
Source: Eur J Hum Genet. 2013 Dec 4;22(7):949–52. doi: 10.1038/ejhg.2013.274 (PMC4060122; doi:10.1038/ejhg.2013.274)
Supplement: Supplementary Figure Legends [file ejhg2013274x3.doc]

**Supplementary Figure 1. GWAS Central content representing a single study.** The study top-level summary data includes panel/cohort information, phenotype summary, details of the genotyping experiments performed, analysis methods and the association result sets. The phenotype data details contain the free-text description and ontology derived annotation for the phenotype(s) in the study, along with the methods undertaken to determine them. The marker data details include the current genomic coordinates for each marker in the study’s association result set and other pertinent information. Data levels are interlinked and also link out to external resources e.g. PubMed, MeSH, HPO and dbSNP.

**Supplementary Figure 2. Distribution of GWAS Central association data across broad MeSH disease categories.** The majority of association data sets are assigned to common disease areas.
